# Epidemiology and cost analysis for patients with oral cancer in a university hospital in China

**DOI:** 10.1186/1471-2458-10-196

**Published:** 2010-04-16

**Authors:** Sheng Han, Yan Chen, Xu Ge, Ming Zhang, Jinwei Wang, Qingbo Zhao, Jianjun He, Zhenghong Wang

**Affiliations:** 1 Department of Information Center, School of Stomatology, the Fourth Military Medical University, Xi'an PR China; 2 Department of Oncology, Xi'Jing Hospital, the Fourth Military Medical University, Xi'an PR China; 3 Administration of Clinical Research and Teaching, School of Stomatology, the Fourth Military Medical University, Xi'an PR China; 4 Department of Logistics, School of Stomatology, the Fourth Military Medical University, Xi'an PR China; 5 Department of physics and mathematics, the Fourth Military Medical University, Xi'an PR China; 6 Training Department, the Fourth Military Medical University, Xi'an PR China

## Abstract

**Background:**

Although several studies have reported the direct cost of oral cancer (OC), little research has invested the factors that could influence the costs of OC patient. This study analyzes the epidemiological characteristics and the direct cost of OC. More specifically, the study examines the relationship between patients' medical costs and influencing factors of epidemiology.

**Methods:**

All patients encountered from January 2007 to December 2007 at the School of Stomatology of the Fourth Military Medical University (FMMU) in China with diagnosis of oral cancer have been selected. Medical hospitalization days (MHD) and cost per patient (CPP) of the samples have been calculated for different patient groups, and the results have been compared using statistical methods.

**Results:**

A total of 456 oral cancer patients have been selected in this study. The epidemical characteristics are as follows: female/male 176/280; squamous cell carcinoma (SCC)/adenocarcinoma/sarcoma/lymphoma/other types 246/127/40/27/16; stage I/II/III/IV 90/148/103/115; smoker/non-smoker 136/320; rural/urban patients 82/374. Of all the patients, 82.24% were over 40 years of age. Rural patients were significantly younger than urban patients. SCC was the majority histology in older patients, while sarcoma was more common in younger patients. 372 of the patients received treatment and 84 gave up any treatment after diagnosis. Treatment cost accounted for majority of the payment. The CPP and MHD of patients in late clinical stage were higher than that of patient in early stage.

**Conclusion:**

Gender, smoking habit and age older than 40 years are the epidemiological risk factors for oral cancer. Lack of medicare, smoking habit, late clinical stage and SCC are the high economic factors for patient medical cost.

## Background

Oral cancer has been recognized as a huge threat to public health because of its high morbidity and mortality. It is estimated that each year there are over 484,000 people diagnosed with oral cancer in the world and approximately 261,000 people die of this disease [1]. In China, over 11,900 cases of oral cancers are diagnosed each year and approximately 5,000 patients die of the disease [1]. A number of factors are associated with the increase of risks of oral cancer. The risk factors include age, tobacco and alcohol consumption, human papilloma virus infection, and race, etc [2-4].

Primary treatments of oral cancer include surgery, radiation and chemotherapy [3,5,6]. These treatments can be employed alone or in combination depending on the clinical stage and histology of oral cancer. Beside primary treatments, oral cancer patients may require additional care to ameliorate the side effects of treatment, such as oral pain due to the tumor or oral mucositis, weight loss, fatigue, nausea, vomiting, and altered salivary gland function [7,8]. Owing to the increasing morbidity and mortality of oral cancer, the treatment costs increase fast. In China, the costs for treating oral cancer bring a heavy financial burden to both social resources and patient's family. On top of direct cost for diagnosing, treatment, and hospitalization, indirect cost includes loss of productivity of patients due to morbidity and disability is very difficult to define and calculate. Given that a patient's medical bill does not reflect all the cost of his or her treatment, in this study, we chose to use inpatients' medical bill as major evidence to indicate the rough cost of their treatment [9]. The information of patients' direct costs can be retrieved from the database of the hospital's information system, which archives the detailed medical cost, doctors' administrations, and case files. To date, few studies have been conducted to assess the medical cost for oral cancer treatment and its relationship with the increasing risks of oral cancer.

This study examines the direct cost of treatment of oral cancer in different stages and analyzes the relationship between patients' medical costs and the related factors including gender, age, clinical stage, pathology, and smoking habit. Furthermore, this study also analyzes the factors of medicare and census register because they can enable us to better understand the economic pressure to patients and also help medical administrators, doctors, and patients to make care plans and treatment decisions appropriate to the needs of patients and their families.

## Methods

### Patients

The study included two parts: epidemiological study and cost analysis. All samples for this study were selected from the Stomatology Hospital of FMMU in China. The data were retrieved from the database of this hospital. The observation period for each patient was 12 months, from January to December 2007. The patients were observed from their first visit to the hospital until the end of year. Epidemiological study included the patients who were diagnosed as having malignancies of the oral cavity including tongue, floor of the mouth, buccal mucosa, gingival tissues, retromolar trigon, palate, and lips with pathological evidences. Cost analysis study included those who had received at least one of the following treatments: surgery, chemotherapy and radiotherapy. Patients who gave up treatment in the Stomatology Hospital of FMMU or who chose to receive treatment in other hospitals were not included in the cost analysis study. This study was approved by the Ethics Committee of the School of Stomatology of the Fourth Military Medical University.

### Data Analysis

Data for epidemiological study included age, gender, pathology, clinical stage, and smoking habits. Data for cost analysis included the cost for diagnosing, treatment, and hospitalization. Diagnosing cost encompasses pathology, radiation, and laboratory testing; treatments cost includes surgery, chemotherapy, radiotherapy, and concomitant medications; and hospitalizations cost goes to nursing, supportive care and lodging.

The cost was calculated in Chinese Yuan (RMB). The charge for every item of service and medication was set by the Price Administration Bureau, and the fees were similar in same grade hospitals as the FMMU hospital. Considering there was no subsidy in the billing record in the FMMU hospital, there was no need to fix the cost bias as had to be done in some other studies.

### Statistical analysis

Continuous variables are presented with mean and standard deviation, while discrete variables are presented with absolute and relative frequencies. Chi-square tests were used for the comparisons of proportions. Student's t-tests were computed for the comparison of mean values. Differences on CPP(cost per patient) and MHD(medical hospital days) according pathology were determined by analysis of variance(ANOVA). The differences on the costs of diagnose, treatment and inpatient according pathology were also determined by ANOVA. Risk factors for squamous cell carcinoma (SCC) were determined by analysis of logistic regression while the variables for CPP were determined by multiple liner regression. Adjusted odds ratios with 95% confidence intervals were computed from the results of the logistic regression analyses. All p-values reported are two-tailed. Statistical significance was set at 0.05 and SPSS 17.0 was employed to perform all of the analyses.

## Results

### Epidemiology

During the period of 2007, Stomatology Hospital of the FMMU received a total of 456 new patients (176 females, 280 males) that had been previously diagnosed with oral cancer. The pathologies of this group of samples included SCC, lymphoma, adenocarcinoma, sarcoma and others. Cytoma, small cell carcinoma, melanoma and clear-cell carcinoma were put into "others" category for their rarity. Of the 456 oral cancer patients, 53.9% (246/456) were SCC; 27.85% (127/456) were adenocarcinoma; 8.77% (40/456) were sarcoma; and 5.92% (27/456) were lymphoma. According to the TNM stage (WHO for Staging Oral Cancer), 32.46% (148/456) were diagnosed at stage II, 25.21% (115/456) at stage IV, 22.59% (103/456) at stage III and 19.74% (90/456) at stage I. 29.82%(136/456) patient had smoking habit while 132 were male and 4 were female (table 1). Of the 136 smoking patients, 69.85% (95/136) got SCC, 17.65% (24/136) got adenocarcinoma, and 12.50% had other pathology types such as lymphoma, sarcoma.

**Table 1:** Sample characteristics

	n	%
**Gender**		
Male	280	61.40
female	176	38.60
**Clinic stage**		
I	90	19.74
II	148	32.46
III	103	22.59
IV	115	25.21
**Smoking status**		
Yes	136	29.82
No	320	70.18
**Pathology**		
SCC	246	53.95
Adenocarcinoma	127	27.85
Sarcoma	40	8.77
Lymphoma	27	5.92
others	16	3.51
**Census register**		
urban	374	82.02
rural	82	17.98
**Receive any treatment**		
yes	372	81.58
no	84	18.42

**Figure 1:**
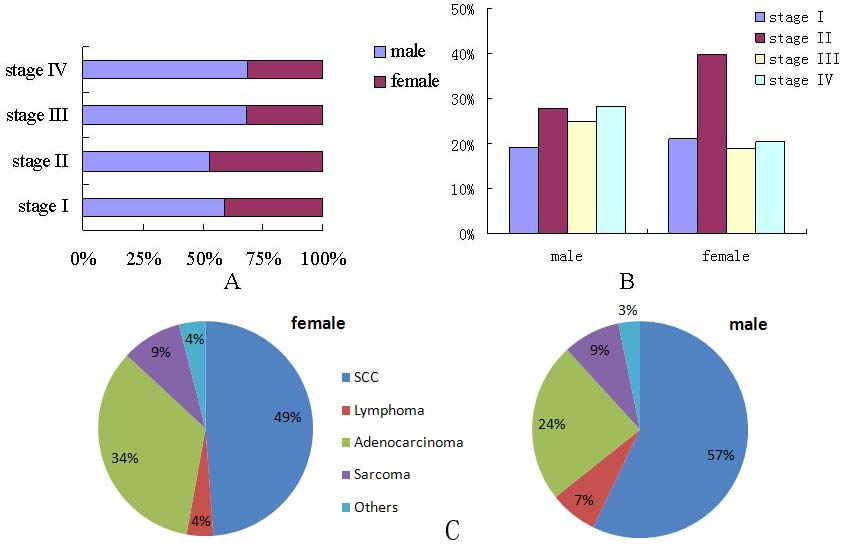
**proportion of gender clinic stage and pathology proportion in the sample data**. Figure 1A gender distribution proportion in different stage. Figure 1B stage distribution proportion in male and female group respectively. Figure 1C pathology distribution in male and female group respectively.

This study also analyzed the gender distribution in different clinical stages (figure 1A). In stage I and II, the proportion of male and female were comparatively even (58.89% vs. 41.11%, and 52.7% vs. 47.3% respectively). But in stage III and IV, the proportion of male was obviously higher than female (67.96% vs. 32.04%, and 68.7% vs. 31.3% respectively). The stage distribution features in male and female groups were also different (figure 1B). Male distribution in each stage was comparatively even (19%, 28%, 25%, 28%), while a majority of female patients distributed in stage II (40%). Findings also indicated that SCC and adenocarcinoma were the most common pathologies in both male and female group (figure 1C). The proportion of adenocarcinoma in female group was higher than that in male group (34.09% vs. 23.93%), while the proportion of SCC in female group was lower than that of male group (48.86% vs. 57.14%).

**Figure 2:**
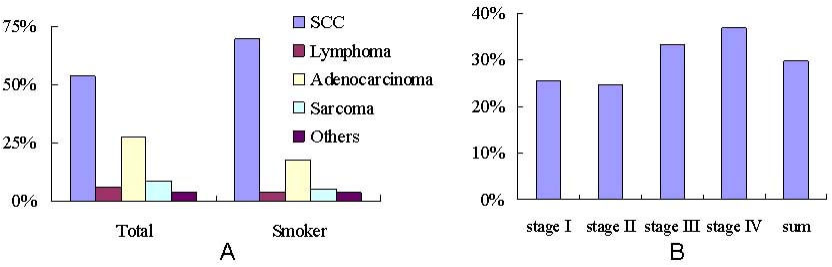
**proportion of smoking patient in the sample**. Figure 2A pathology distribution in the total sample and smoker group respectively. Figure 2B clinical stage distribution proportion in the smoker group.

As tobacco smoking is the most important risk factor for oral cancer, the pathology distribution was examined in the total sample and smoker group respectively (Figure 2). Compared with the total sample, the proportion of SCC in smoker group was relatively high compared with other the non-smoker samples (69.85% vs. 53.95%), while adenocarcinoma, lymphoma and sarcoma shared the low proportions (17.65% vs. 27.85%; 3.68% vs. 5.92%; 5.14% vs. 8.77% respectively) (figure 2A). Table 2 showed that the proportion of male smokers in late stage (III and IV) was evidently higher than that in early stages (I and II) (figure 2B) and the proportion of male smoker with SCC was evidently higher than that with adenocarcinoma.

**Table 2:** The results of comparison of proportion of male smoker in different clinic stage and pathology with the chi-square test

	Smoking status				*p*
	No		Yes		
	Sample	Percent%	Sample	Percent%	**χ**^2 ^**test**
**stage**					0.019
I,II	79	60.31	52	39.69	
III,IV	69	46.31	80	53.69	
**Pathology**					0.001
SCC	67	41.86	93	58.14	
Adenocarcinoma	44	65.67	23	34.33	

Note: Female patient were not calculated because there were only 4 female smokers in the sample. For the same reason, only male patient with SCC or adenocarcinoma were compared in the table.

**Figure 3:**
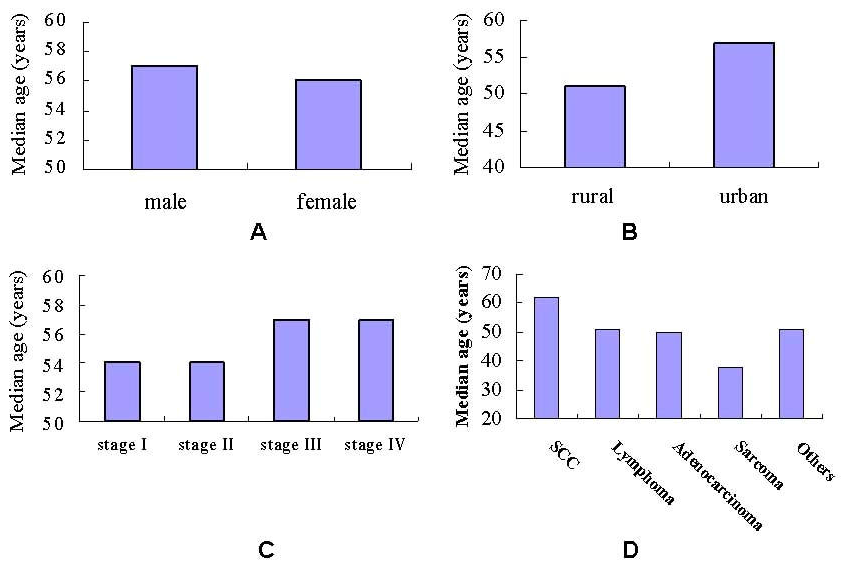
**media age in different groups**. Figure 3A median age of male and female group. Figure 3B median age of rural and urban group. Figure 3C median age in different clinical stages. Figure 3D median age in different pathology types.

The mean age of the total 456 samples was 54.63 ± 16.73 years (range 1-90 years). There was no evident difference between male and female group about mean age (Figure 3A and Table 3). Of the 456 patients, 17.98% (82/456) patients came from rural areas, and 82.02% (374/456) came from urban. The mean age of the rural and urban patients was 48.56 ± 16.79 and 55.94 ± 16.54 respectively, indicating a significant statistical difference (p < 0.01) (figure 3B and Table 3). The mean age of early and late clinical stages showed no significant difference (Figure 3C and Table 3).

**Table 3:** The results of comparison of mean age in different groups with t-test

	**± S**	*p*	n
**Gender**			
male	55.06 ± 17.15	0.598	280
female	54.08 ± 16.26		176
**Rural/Urban**			
Rural	48.56 ± 16.79	0.000	82
Urban	55.94 ± 16.54		374
**Stage**			
I,II	54.51 ± 16.31	0.823	238
III,IV	54.87 ± 17.35		218

We have further probed into the relationship between different age ranges and pathology (Table 4). The total samples were divided into four age range groups: < 20 years, 20~39 years, 40~59 years, and ≥ 60 years. Nearly 82.24% (375/456) patients were older than 40 years of age, and 3.07% (14/456) were under 20 years of age. The proportion of SCC was higher in the age group of over 59 but rare in the young group (< 20 years). The proportion of adenocarcinoma was relatively higher in the age group of 40-59 (48.83%).

**Table 4:** Age distribution in different pathology

Age range	n(%)				
	<20	20~	40~	>60	total
**Pathology**					
sarcoma	8(20)	13(32.5)	12(30)	7 (17.5)	40(100)
SCC	0(0)	19(7.72)	88(35.77)	139(56.51)	246(100)
lymphoma	4(14.81)	5 (18.52)	7(25.93)	11(40.74)	27(100)
adenocarcinoma	2(1.57)	29(22.83)	62(48.83)	34(26.77)	127(100)

Table 5 showed the logistic regression analysis of independent factors of SCC and adenocarcinoma. The results showed that smoking habit and age were independently associated with SCC. Patients with smoking habit had 3.032 times greater odds (95%CI: 1.565-5.872) for SCC than non-smoking patients. The odds for SCC were 3.231 times higher for patients older than 40 compared with patients younger than 40 years old.

**Table 5:** results of logistic regression that evaluate age, gender, smoking habit and census register factors in relation to SCC

	OR(95% CI)	*p*
Gender	1.164(0.675-2.008)	0.5854
Smoking habit	3.032(1.565-5.872)	0.0010
Age	3.231(1.622-6.436)	0.0009
Census register	1.020(0.570-1.827)	0.9457

Note: only SCC and adenocarcinoma patient was selected for the analysis because of the rarity of the others pathology groups.

### Cost Analysis

The primary goal of cost analysis in the study was to identify the relationship of differences in MHD and CPP with pathology, clinical stage, gender, smoking habit, medicare and census register.

Of the all the patients selected to this study, 372 patients had received at least one of the three treatments, surgery, radiation and chemotherapy, and 84 patients chose not to receive any treatment in this hospital. 89.20% of those who received treatments in this hospital did not have medicare (332/372); only 10.80% of them (40/372) were covered with medicare.

#### Gender, medicare, rural/urban factor, smoking habit

**Figure 4:**
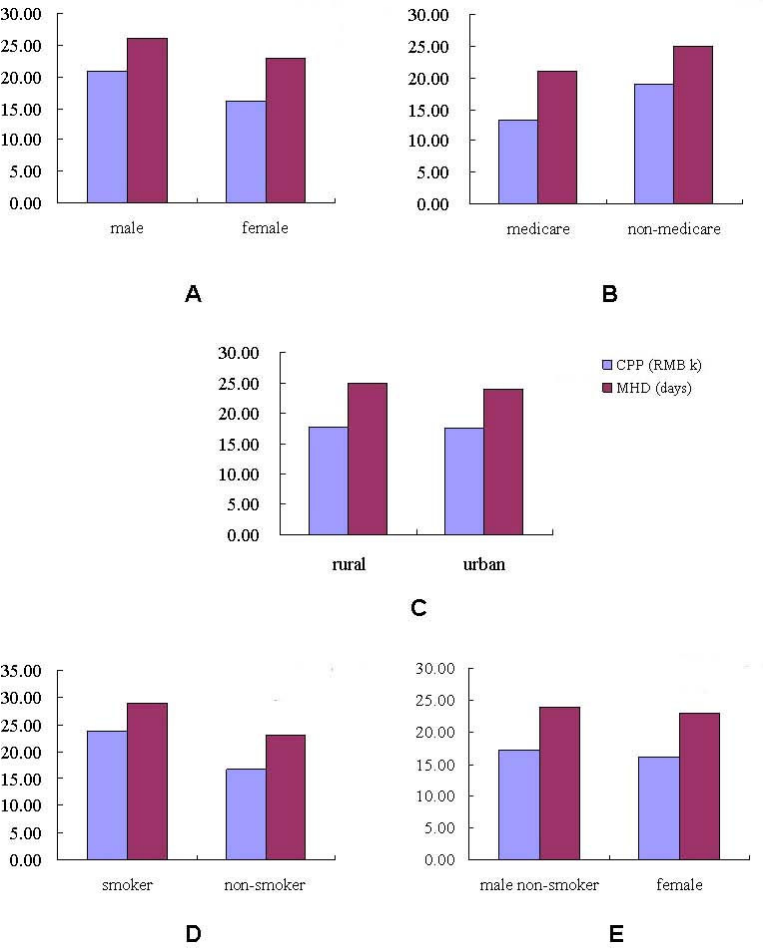
**MHD and CPP associated with gender, medicare, censue register and smoking habit**.

Statistical results indicated no significant difference between the MHD of male and female (*p *= 0.19). However, the CPP of male was significantly higher than that of female (*p *= 0.039) (Table 6, Figure 4A). No significant difference was found in the MHD between the patients with medicare and those without medicare (*p *= 0.628). Interestingly, the CPP of those with medicare were evidently lower than those without medicare (*p *= 0.002) (Table 6, Figure 4B). This study has also found that whether the patients came from the country or the cities, the MHD and CPP showed no evident difference (Table 6, Figure 4C).

**Table 6:** The result of comparison of MHD and CPP in different groups with t-test

	MHD(days)		CPP(RMB)	
	± S	*p*	± S	*p*
**Gender**				
male	32.31 ± 11.22	0.19	25890.46 ± 9663.03	0.039
female	29.55 ± 10.74		22071.86 ± 8537.38	
**Smoking**				
smoker	35.07 ± 11.89	0.018	27580.14 ± 9721.63	0.025
non-smoker	29.67 ± 10.60		23085.26 ± 9174.67	
male non-smoker	29.86 ± 11.35	0.898	24149.55 ± 9284.89	0.326
female	29.55 ± 10.74		22071.86 ± 8537.38	
**Medicare**				
medicare	29.84 ± 9.97	0.628	18217.54 ± 8933.08	0.002
non-medicare	31.39 ± 11.91		25193.69 ± 9652.72	
**Censue register**				
rural	31.44 ± 10.17	0.896	24165.41 ± 8141.78	0.901
urban	31.12 ± 9.78		24425.96 ± 8424.43	
**Stage**				
I,II	27.25 ± 10.74	0.000	19544.09 ± 7412.78	0.000
III,IV	35.96 ± 12.67		30163.82 ± 9039.12	

Smoking habit is a crucial factor in the development and prognosis in oral cancer. Therefore, this study sought to ascertain whether or not smoking habit could affect the direct cost for this disease. Table 6 and Figure 4D demonstrate that the MHD and CPP of smokers were significantly higher than those of non-smokers (p = 0.018). Considering the higher proportion of smokers in the male than female group, the MHD and CPP of patients in male non-smoker samples were compared with the female group. The MHD and CPP in the two groups showed no evident difference (*p *= 0.898, *p *= 0.326) (Table 6 and Figure 4E).

**Figure 5:**
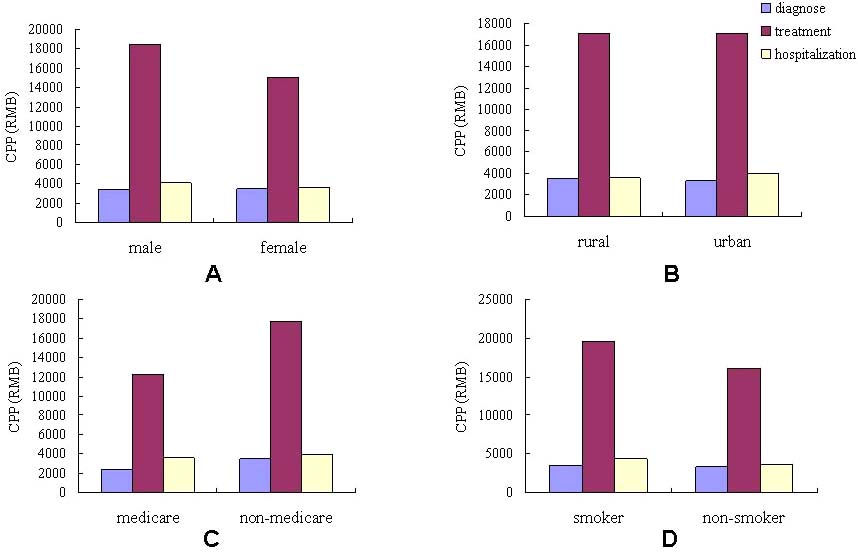
**Diagnose, treatment and hospitalization cost associated with gender, censue register, medicare and smoking habit**.

The total cost for oral cancer treatment is comprised of three parts: diagnosis, treatment and hospitalization. Figure 5 indicates that treatment accounts for the major cost. In terms of overall cost, there was no difference between rural patients and urban patients (Figure 5B and Table 7). Table 7 also indicates that the cost for diagnosis and hospitalization in both gender groups had no significant difference (*p *> 0.05), however, the treatment cost of the male patients was significantly higher than the female patients (*p *= 0.015) (Figure 5A, Table 7). Although, there was no evident difference between patients with medicare and without medicare in hospitalization, it is noticeable that the diagnosing and treatment cost for patients with medicare was significantly lower than those without medicare (Figure 5C, Table 7). Comparing the cost of smokers with non-smokers, there was no difference in the cost diagnosing between these two groups, however, the smokers had significantly higher cost for treatment and hospitalization than those non-smokers, (Figure 5D, Table 7).

**Table 7:** The result of comparison of mean cost of diagnosis, treatment and hospitalization in different groups with t-test

	Diagnosis		Treatment		Hospitalization	
	± S	*p*	± S	*p*	± S	*p*
**Gender**						
male	3366.87 ± 1200.11	0.78	18437.64 ± 7680.07	0.015	4085.95 ± 1331.44	0.071
female	3472.55 ± 1330.50		15034.64 ± 6947.31		3564.67 ± 1199.15	
**Censue register**						
rural	3484.04 ± 880.37	0.816	17054.52 ± 8800.5	0.987	3626.85 ± 1288.04	0.309
urban	3384.34 ± 1119.73		17081.32 ± 8159.8		3960.31 ± 1522.72	
**Medicare**						
yes	2360.03 ± 1165.69	0.000	12294.71 ± 6546.36	0.002	3562.81 ± 1141.77	0.414
no	3551.40 ± 1277.87		17722.52 ± 8625.68		3955.21 ± 1407.36	
**Smoking**						
yes	3502.53 ± 1056.91	0.751	19632.98 ± 7443.40	0.023	4444.63 ± 1402.97	0.011
no	3372.21 ± 1219.56		16060.77 ± 7175.36		3652.28 ± 1263.66	
**Stage**						
I,II	2814.56 ± 795.47	0.000	13396.73 ± 7596.94	0.000	3332.8 ± 1386.07	0.000
III,IV	4125.54 ± 964.92		21505.33 ± 8272.4		4532.95 ± 1706.4	

#### Stage and Pathology

We examined the detailed MHD and CPP of patients in different clinical stage and with different pathology. Table 6 indicate that the MHD and CPP of patients in early clinical stages (I~II) were lower than those in late stages (III~IV). Table 8 showed the comparison of MHD and CPP between different pathology groups. The CPP and MHD of adenocarcinoma group were evidently lower than that of SCC and sarcoma group. There were no significant different in MHD and CPP between SCC and sarcoma group.

**Table 8:** ANOVA analysis of MHD and CPP with different pathology

	MHD(days)		CPP(RMB)	
	± S	ANOVA	± S	ANOVA
**Pathology**		F = 14.865 *p *= 0.000		F = 9.643 *p *= 0.000
SCC	35.24 ± 13	#	27889.93 ± 11031.95	#
sarcoma	38.27 ± 12.81	#	27635.03 ± 10647.21	#
adenocarcinoma	23.61 ± 10.27	* ^	19222.25 ± 9222.52	* ^

Note: lymphoma and other group were not calculated for their rarity

*: significant different with SCC

^: significant different with sarcoma

#: significant different with adenocarcinoma

**Figure 6:**
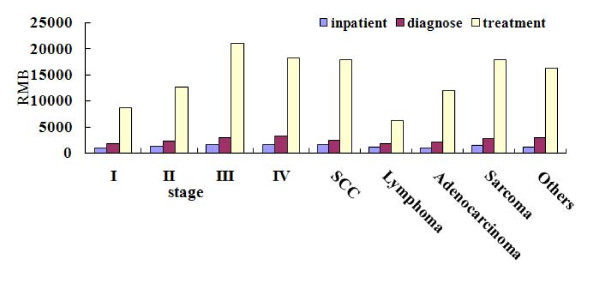
**Diagnose, treatment and hospitalization cost associated with clinical stage and pathology**.

The study has further analyzed the costs for diagnosis, treatment and hospitalization in different clinical stages (Figure 6A, Table 7). The findings showed that the cost for diagnosing, treatment and hospitalization in early stage were significantly lower than those in late stage (Table 7).

The data indicated that the cost for diagnosis in each pathology group had no evident differences (p > 0.05). The treatment cost in adenocarcinoma group was significantly lower than that of SCC groups. The hospitalization cost of adenocarcinoma group was significantly lower than that of SCC and sarcoma group. (Figure 6B, Table 9).

**Table 9:** ANOVA analysis of cost of diagnosis, treatment, and hospitalization in different pathology

	Diagnosis		Treatment		hospitalization	
	± S	ANOVA	± S	ANOVA	± S	ANOVA
Pathology		F = 0.006 *p *= 0.994		F = 10.831 *p *= 0.000		F = 12.746 *p *= 0.000
SCC	3465.22 ± 1058.89		19995.22 ± 9700.83	#	4429.49 ± 1618.05	**#**
sarcoma	3437.07 ± 1027.85		17064.89 ± 7305.06		4309.36 ± 1026.71	**#**
adenocarcinoma	3507.06 ± 1221.29		12787.87 ± 4798.98	*	2927.32 ± 683.41	* ^

Note: lymphoma and other group were not calculated for their rarity

*: significant different with SCC

^: significant different with sarcoma

#: significant different with adenocarcinoma

#### Variables for cost

To determine the independent variables for the costs of OC patient, multiple regression was made. Table 10 showed that medicare, pathology and clinical stage were associated with the cost of OC patient. Age, smoking habit, gender and census register were not the independent variables for the cost of OC patient.

**Table 10:** Results of multiple regression that evaluate age, gender, medicare, smoking habit, pathology, census register variables in relation to costs of OC patients

	Regression Coefficient	Standard Error	*p*
Intercept	6852.744	4327.053	0.1143
Age	3056.266	2741.573	0.2658
Medicare	4851.098	2903.071	0.0457
Gender	2349.413	2183.264	0.2827
Smoking habit	62.7796	2384.648	0.9790
Pathology	7420.790	1981.564	0.0002
Census register	241.7844	2210.423	0.9130
Clinic stage	10154.28	1885.294	0.0000

Design of variable

Age: 0 = younger than 40 years old, 1 = older than 40 years old

Medicare: 0 = medicare, 1 = without medicare

Gender: 0 = female, 1 = male

Smoking habit: 0 = no, 1 = yes

Pathology: 0 = adenocarcinoma, 1 = SCC

Census register: 0 = rural, 1 = urban

Clinic stage: 0 = early stage, 1 = late stage

Note: only SCC and adenocarcinoma patient was selected for the analysis because of the rare sample of the other pathology groups.

## Discussion

### Epidemiological Characteristics and Risk Factors

According to report that the incidence of oral and pharyngeal cancer is increasing to be the 8th most common malignancy worldwide [1]. Numerous studies have already identified cigarette smoking as an important factor to the cause oral cancer [3,10-12]. This study has also confirmed the relationship between SCC and smoking. Although the proportion of SCC in the total sample was the highest (53.95%), it was particularly higher in the smoker group, accounting for nearly 70%. Our study indicates that smoking habit significantly correlates the smoking patients to late clinical stage. The proportion of smoking patients in late stage was higher than that in early stage, and this may mean poor prognosis [12]. Therefore, the high risk factor of cigarette smoking should be widely acknowledged.

The result of this study has shown the occurrence of oral cancer in male population was higher than in female population. Males were more affected by oral cancer because of their exposure to carcinogenic factors associated with this tumor, such as tobacco and alcohol [3,10-12]. No median age differences existed between men and women, and this is in agreement with previous reports [13]. Regarding the distribution of pathology over the clinical stages, the incidence of male in every stage, particularly in late state, was higher than that of female. Compared with the male patients, the female patients were more diagnosed in early stage (*p *= 0.004), indicating better prognosis. Although SCC and adenocarcinoma were the most common pathologies in both male and female groups, the proportion of SCC in female was lower than in male group. This phenomenon can probably also be explained by smoking habits.

The age information in the sample patients indicates that oral cancer is more common among the elderly. Of the 456 patients, 196 were over 60 years of age; only 14 were under 20 years of age. The patients under 40 years of age were less than 1/5 of the total, while those over 40 were more than 4/5 of the total. This result, which is similar to the results reported in other studies [14], indicates that doctors should pay more attention to patients over 40 years of age and suggest that these patients receive oral examination on a regular basis. Regular oral examination can help identify oral cancer at early stage. Treatment delivered at early oral cancer stage can improve patients' survival opportunity and reduce their medical expenses.

Some investigators have reported poor prognosis for older patients [15-17] and came to the conclusion that late clinical stage means poor prognosis. In our study, we analyzed the relationship between clinical stage and median age. The result has shown that there were no significant differences between early and late clinical stage groups. The study has also found that SCC was more common in older patients, that is, confirming the association with older patients and the higher incidence of SCC. This may also be related to smoking, because cigarette smoking was more prevalent in the older-age group and has resulted in a significantly increased risk of SCC in many tissues and organs such as oral, lung and esophagus [18]. However, the incidence of sarcoma decreased with the increase of age, and this may be related to the high incidence of soft tissue carcinoma in younger people [19,20]. It seems that adenocarcinoma mainly exists in middle age patients, and the reason for this remains to be explored in future studies.

The mean age of rural patients was significantly younger than that of urban patients. This may be related to the oral hygienic habits in rural areas. For example, a recent study of 103 oral cancer patients in Russia reveals that people living in the country had fewer teeth-brushing on a daily basis than people living in the city, and rural patients had oral cancer at an earlier age than urban patients [21]. The results of this study support this evidence, but the high oral cancer risk at earlier ages among rural populations is probably related not only to the poor hygienic habits of rural people but also to the poor hygienic environment of the rural area. Owing to poverty and lack of clean water, some rural residents in the west China may experience difficulty with good oral hygienic habits. In future research, more efforts should be directed to rural hygienic environment and personal oral hygienic habits. Lack of knowledge about oral cancer also increases the risk of oral cancer development. Although oral cancer can easily be found by patients through a personal oral examination, many patients ignore it and some doctors do not pay enough attention [22]. Doctors' ignorance of oral cancer can often result in a delay of an official oral cancer diagnosis. Based upon this information, it is necessary to introduce more knowledge of oral cancer to both ordinary people and health care providers.

The logistic regression analysis showed the smoking habit and age were the independent risk factors for SCC (Table 5). Although gender was not the independent risk factor in the analysis, it should still be noticed for almost all smokers of the study were male. The proportion of female smoker was very low in china, so the gender factor should also be paid attention by the dentist.

### Economic characteristics and related factors

In China, with the increasing cost for cancer, health care administrators and researchers have paid more attention to healthcare economics. In recent years, some researchers have investigated the cost related to clinical stage, age, etc. of oral cancer [23-25]. However, these studies did not include other contributory cost factors such as gender, smoking habit, and pathology. The aim of this study is to find and analyze the epidemiological characteristics and direct medical costs of oral cancer in China. Findings from this study support and further enhance previous findings on the cost of treating oral cancer.

An earlier study has reported that non-smoker patients at any age may have a worse clinical outcome, and the researchers suggested that this group of patients be evaluated for unique genetic and environmental etiologies, and should probably be approached in a more aggressive fashion [26]. However, the result of our study came to a different conclusion. The cost differences mainly existed in treatment and hospitalization but not in diagnosis. There was no difference in MHD and CPP between male non-smokers and female non-smoker groups; however, the MHD and CPP of smokers were significantly higher than those of non-smokers. This result indicates that clinical outcomes are more related to smoking habit of oral patients than to gender.

The treatment cost for male patients was significantly higher than female patients although the MHD was comparative. This may have two explanations. One explanation is that the female patients were diagnosed more frequently in early clinical stage than male patients. The findings of two studies respectively conducted in Greece and the United States support this observation that the average cost in late stages (III~IV) was evidently higher than that in early stages (I~II)[23,25]. This study has also confirmed this conclusion. In the current study, the CPP in late stages was nearly twice more than that in early stages, and the MHD in late stages was also longer than that in early stages. The high cost for treatment is related to the advanced stage of cancer because patients need more extensive and aggressive treatment. Therefore, early detection may increase diagnose rate, and early treatment may decrease the total cost for oral cancer patients.

The other explanation for a higher cost of male oral cancer patients is the higher proportion of SCC in male patients than in female patients. We make this new argument based on our study and analysis of the samples of different pathology. Within the two main types of oral cancer (SCC, adenocarcinoma) in this study, the CPP of SCC was higher, and the CPP differed mainly in treatment and hospitalization. This can partially explain why the cost for female patients was lower than that of male patients.

What is more, the analysis in this study has also included cost differences between patients with and without medicare and between rural patients and urban patients. According to our study, the cost of the rural patients was similar to that of the urban patients, but the former paid more because of the low coverage rate of medicare in rural areas in China. The coverage of medicare in China has been expanded fast in recent years; however, there are still a lot of patients, particularly in rural areas, who had to afford their medical treatment by themselves. The burden of medical bill for oral cancer treatment was heavier to patients in rural than in urban. Our data showed that 12.53% of the urban patients were covered by medicare while only 1.23% of the rural patients were supported with medicare. Further analysis has revealed that the cost difference mainly existed in diagnose and treatment, but not in hospitalization. In China, some examinations, drugs and treatment measures are not covered by medicare, and the costs incurred by these items should be paid by patients themselves. In order to reduce this kind of costs, doctors and patients would tend to choose those items covered by medicare. For non-medicare patients, their examination and treatment were not limited by medicare regulations, so doctors and patients would choose the most effective and safe items to diagnose and treat oral cancer, and thus would directly cause the increase of medical costs. Although the medicare coverage list expands in recent years, more sophisticated and comprehensive treatment and more effective medicines should be added on the medicare list.

The result of multiple regression showed that the smoking habit was not the independent factor for the cost of OC patient. But the proportion of smoker in late stage was higher than that of early stage and the proportion of smoker in SCC was obviously higher than other group. These results indicated that smoking were associated with stage and pathology of OC and would associate with the cost of OC patient indirectly.

## Conclusion

Although this study has not explored many other factors such as pathological grade, longer time cost analysis, survival rate and so on, which need to be further studied, the following conclusions may be drawn: (1) Smoking is an important risk factor that is not only related to development of oral cancer, but also increases the total cost and medical hospitalization days for oral cancer patients; (2) Gender, smoking habit and age are the related factors for oral cancer; (3) The median age of rural patients was younger than that of urban patients; (4) The proportion and cost for SCC were higher than those of adenocarcinoma. (5) The MHD and CPP in late clinical stage were higher than those in early stage. (6) The CPP of patients without medicare was higher than those with medicare.

To reduce the economic loss to oral cancer patients and to social resources, it is crucial that oral cancer can be identified and treated at its early stage. Physicians must be aware of the possibility of oral cancer, particularly among those with high risk factors and premalignant oral lesions such as leukoplakia. To reduce morbidity and mortality attributable to oral cancer, greater efforts should be made for prevention. Primary prevention of oral cancer includes avoidance of tobacco use and alcohol abuse, appropriate intake of fruit and vegetables, and good oral hygienic habits. In addition, people should be routinely screened for oral mucosal lesions, which is essential for early detection.

## Competing interests

The authors declare that they have no competing interests.

## Authors' contributions

SH, YC and XG conducted the study and drafted the report. JW, MZ, JH, ZW and QZ performed the statistical analysis and data collection. All authors read and approved the final manuscript.

## Pre-publication history

The pre-publication history for this paper can be accessed here:

http://www.biomedcentral.com/1471-2458/10/196/prepub
